# MYC and MiR: vicious circle

**DOI:** 10.18632/oncotarget.1602

**Published:** 2013-11-28

**Authors:** Xiaohong Zhao, Xinwei Zhang, Jianguo Tao

**Affiliations:** Departments of Hematopathology and Laboratory Medicine, H Lee Moffitt Cancer Center and Research Institute at the University of South Florida, Tampa, FL, USA

Increasing evidence now suggests that a dynamic interaction occurs between the lymphoma cell and its microenvironment (TME, stroma), with each profoundly influencing the behavior of the other. The TME is a critical determinant for tumor initiation, progression, response to therapy, and drug resistance [1, 2]. Specific niches within the lymphoma TME provide a sanctuary for subpopulations of lymphoma cells and provide a survival advantage through stroma–lymphoma cell interaction. This interaction further contributes to the resistance of residual lymphoma cells to chemotherapeutic agents, and the residual lymphoma cells are then destined to expand and evolve through acquisition of additional genetic abnormalities (or selection of pre-existing clones) that lead to relapse and progression of lymphoma [3]. Therefore, TME are important determinants contributing to minimal residual disease and drug resistance, a problem that remains a major challenge in the treatment of B-cell malignancies. However, how the lymphoma TME influences lymphoma cell survival, response to therapy and the molecular mechanisms involved remains unclear. Our group and others have demonstrated that B-cell lymphoma is a disease that depends on the strong interactions between B cells and TME [4-6]. Our previous studies have shown that adhesion of lymphoma cells to lymph node and bone marrow stromal cells (HK, HS-5) results in inhibition of cell apoptosis upon exposure to chemotherapeutic drugs in a variety of B cell malignancies[4]. Recently, by combining traditional monolayer co-culture with colony formation technique, a novel TME co-culture model, we demonstrated lymphoma stroma cells (HK or HS-5) could alter the anchorage-independent clonogenic growth of lymphoma cells [7]. Next, we utilized the severe combined immunodeficiency (NOD-SCID) mouse model and injected lymphoma cells with or without stromal cells and observed a more robust growth of tumor in mice receiving HK and lymphoma cells[7]. The combination of traditional monolayer co-culture with in vitro colony formation and in vivo tumor formation, creating secondary tumor-like structure, more accurately simulates complex lymphoma TME, thus constitute innovative critical platforms to test anti-lymphoma drugs in the context of TME-mediated lymphoma growth and drug resistance.

When applied these models to determine the functional role of miRNAs and miRNA-regulated proteins in TME-mediated drug resistance and lymphoma progression, a global miRNA expression profiling was performed and revealed that expression of multiple miRNAs is altered in lymphoma cells upon adhesion to HK cells [4]. Among these miRNAs, miR-548 family members miR-548f, miR-548h, and miR-548m were among the most downregulated miRNAs by stroma interaction. To determine whether miR-548m is involved in TME-mediated lymphoma survival and growth, ectopic miR-548m expression was shown to induce cell apoptosis, and overcame stroma-mediated drug resistance. Moreover, transfection of pre-miR-548m resulted in over-expression of miR-548m and significantly abolished stromal cell-induced clonogenic growth ex vivo and dramatically suppressed in vivo lymphoma formation and blocked stroma-induced lymphoma growth. Overall, these results support the key role of miR-548m in TME-mediated lymphoma therapy response and progression.

Next, HDAC6 and MYC were identified as direct down-stream miRNA-548m targets in 3'-untranslated region (UTR)-dependent fashion [7]. On the one hand, HDAC6 was shown to mediate miR-548m function through down-regulation of proapoptotic protein, Bim, conferring lymphoma cell survival and drug resistance. On the other hand, in addition to act as an intermediary for miR-548m, MYC repress the expression of miR-548m by binding to E-box of miR-548m promoter [7], and MYC and miR-548m form a (double negative) feed-forward loop, leading to sustained MYC activation and miR-548m down-regulation and constitute a key determinant for stroma-mediated cell growth and clonogenicity in B-cell lymphomas. Given that MYC overexpression not only promotes lymphoma progression by inducing proliferation but also makes the lymphoma cells prone to apoptosis, a concomitant stroma-activated pro-survival signal pathway is essential to cooperate with MYC in lymphoma TME to confer lymphoma cell survival advantage, drug resistance and proliferation potential. Thus, it is *conceivable* that stroma-induced, miR-548m mediated HDAC6-BIM and MYC pathways cooperatively dictate and promote lymphoma drug resistance and progression (Figure 1). Disruption of both pathways establishes a novel *“double-hit” strategy:* targeting the pathway (HDAC6) related to survival, targeting the pathway (MYC) related to cell proliferation and lymphoma progression. Indeed, we demonstrated that the combination of HDAC6-selective inhibitor tubastatin A and MYC inhibitor, JQ1, in synergy significantly enhances cell death, abolishes TME-mediated drug resistance, and suppresses clonogenicity and lymphoma growth ex vivo *and in vivo* [7]. Together, these data suggest that the lymphoma-stroma interaction in lymphoma TME directly impacts the biology of lymphoma through genetic and epigenetic regulation and with HDAC6 and MYC as potential therapeutic targets.

**Figure 1:**
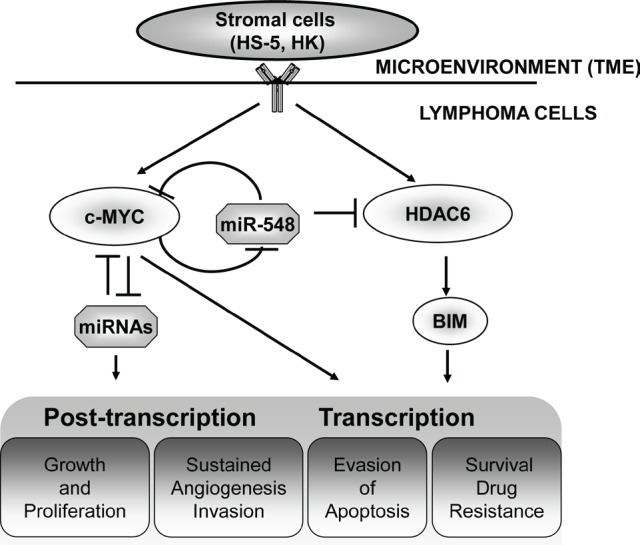
Microenvironment-mediated c-Myc-miR- 548m amplification loop drives drug resistance, clonogenic growth and tumor progression in B-cell lymphomas Microenvironment and lymphoma interaction generates feedback loop(s) to ensure sustained high MYC and low level of miRNA(s) contributing to lymphoma cell survival, growth and progression through transcriptional and post-transcriptional mechanism.

Of another note, the above model underscores the complexity of interaction of lymphoma with TME. The MYC transcriptional network has been shown to include miRNAs[8]. Stroma-activated MYC and miRNAs form interacting network and underlie the molecular mechanism of TME-promoted lymphomagenesis through the transcriptional and post-transcriptional regulation of their target genes involved in cell proliferation, angiogenesis, invasion and suppression of apoptosis (Figure 1). To this end, functional identification of MYC, MYC target genes and other survival signal activation in TME with development of novel pharmacological inhibitors will allow us to gain insights into B-cell lymphoma progression and provide novel biological targets for B-cell malignancy therapy.

## References

[R1] Dalton W S (2004). Semin Hematol.

[R2] Janikova A (2011). Leukemia & lymphoma.

[R3] Meads M B (2009). Nat Rev Cancer.

[R4] Lin J (2011). Leukemia.

[R5] Park C S (2005). Immunology.

[R6] Ame-Thomas P (2007). Blood.

[R7] Lwin T (2013). J Clin Invest.

[R8] Chang T C (2008). Nat Genet.

